# Surviving the Storm: Cardiac Tamponade and Effusive Constrictive Pericarditis Complicated by Pericardial Decompression Syndrome Induced by COVID-19 Infection in the Setting of Newly Diagnosed Acute Myeloid Leukemia (AML)

**DOI:** 10.7759/cureus.56710

**Published:** 2024-03-22

**Authors:** Wassim Abouzeid, Noreen Mirza, Paul Bellafiore, Chrystina Kiwan, Amy Paige, Addi Suleiman, Ahsan Khan, Richard Miller

**Affiliations:** 1 Internal Medicine, Saint Michael's Medical Center, Newark, USA; 2 Pulmonology and Critical Care, Saint Michael's Medical Center, Newark, USA; 3 Cardiology, Saint Michael's Medical Center, Newark, USA

**Keywords:** acute myeloid leukemia (aml), pericardial decompression syndrome, effusive pericarditis, covid-19, pericardial effusion. cardiac tamponade

## Abstract

Coronavirus disease 2019 (COVID-19)-induced pericarditis and pericardial myocarditis are common entities; however, the development of pericardial effusion post-COVID-19 infection has only been reported in about 5% of cases. Rapid and acute progression to pericardial tamponade is uncommon, and progression to effusive constrictive pericarditis (ECP) and pericardial decompression syndrome (PDS) is an even rarer phenomenon. We describe these phenomena in this report to raise awareness and aid clinicians in the early diagnosis and management of these conditions. We report a case of a 45-year-old female with a past medical history of recent COVID-19 infection, uncontrolled diabetes mellitus, and hypertension who presented with severe chest pain, which was determined to be acute pericarditis post-COVID-19 infection. The patient developed a large pericardial effusion leading to cardiac tamponade within one day of initial presentation. Urgent pericardiocentesis was performed but was complicated by rapid decompensation of the patient, which has been assumed to be ECP following pericardiocentesis and PDS.

Close monitoring of acute pericarditis with pericardial effusion is required in these patients for the early detection of cardiac tamponade, which requires urgent pericardiocentesis. Judicious post-pericardiocentesis follow-up is also required for the early diagnosis of conditions such as ECP and PDS. These cases are generally managed symptomatically, but in cases of severe ECP syndrome, pericardial stripping may be required.

## Introduction

Despite the extensive medical research over the past three years since the coronavirus disease 2019 (COVID-19) pandemic outbreak, the exact mechanism of cardiac involvement in COVID-19 infection is poorly understood. Although, pericarditis and pericardial myocarditis have been previously described as common manifestations of cardiac involvement in COVID-19 infections. pericardial effusion accounts for only 5% of cardiac involvement in such conditions. Effusive constrictive pericarditis (ECP) is an even rarer entity pertaining to cardiac involvement in COVID-19 infections [1-3]. We discuss a challenging case of a 45-year-old female with a recent history of mild COVID-19 infection, uncontrolled diabetes mellitus, and hypertension who presented with chest pain and was diagnosed with ECP. This case report describes the clinical presentation, diagnostic workup, and management of this complex condition, as well as the occurrence of pericardial decompression syndrome (PDS).

Non-steroidal anti-inflammatory drugs (NSAIDs) and colchicine are considered the mainstay of treatment in acute pericarditis. Corticosteroids are reserved for cases of treatment failure, resistance, or contraindications to first-line therapy. Furthermore, interleukin-1 (IL-1) receptor antagonists, intravenous immunoglobulins (IVIG), and azathioprine are recommended in refractory recurrent pericarditis patients. Pericardial tamponade in the setting of acute pericarditis is managed with pericardiocentesis and occasionally a pericardial window may be required. Post-pericardiocentesis ECP is common and about 16% of patients who undergo pericardiocentesis may develop this condition, which requires similar therapies including anti-inflammatory medications; it may require subsequent pericardiotomy in up to 16% of cases [4-6].

PDS is an uncommon complication of pericardial drainage but is associated with high mortality and morbidity rates. RV dilatation that leads to TR and cardiogenic shock post-pericardiocentesis is considered the hallmark of PDS. The treatment usually involves supportive management, intending to improve issues with ventricular function, which are observed in most survivors.

## Case presentation

A 45-year-old female with a past medical history of a recent mild COVID-19 infection two weeks before presentation, which, according to the patient, had not required hospital admission or treatment, uncontrolled diabetes mellitus (HbA1c: 11.1), and hypertension presented to our hospital with two days of constant, non-exertional chest pain radiating to her back and relieved with sitting forward. She denied any shortness of breath or palpitations. On examination, her temperature was 98.4 °F, her heart rate was 110 beats per minute, her respiratory rate was 18 breaths per minute, and she was saturating at 98% on room air. Physical examination revealed chest wall and left upper back tenderness with regular S1 and S2. There were no murmurs, rubs, or gallops. The initial laboratory data are shown in Table 1.

**Table 1:** Laboratory data on admission and post-pericardiocentesis

	Admission labs	After pericardiocentesis	Reference range
Sodium	135 mmol/L	131 mmol/L	136-145 mmol/L
Potassium	3.6 mmol/L	4.4 mmol/L	3.5-5.3 mmol/L
Bicarbonate	23 mmol/L	17.0 mmol/L	20-31 mmol/L
Chloride	102 mmol/L	101 mmol/L	98-110 mmol/L
Anion gap	9.4	13	<12
Blood urea nitrogen	9.0 mg/dL	25.0 mg/dL	6-24 mg/dL
Creatinine	0.8 mg/dL	1.4 mg/dL	0.6-1.2 mg/dL
Total bilirubin	0.4 mg/dL	0.6 mg/dL	0.2–1.2 mg/dL
Aspartate aminotransferase	17 U/L	82 U/L	10-36 U/L
Alanine aminotransferase	23 U/L	72 U/L	9-46 U/L
Alkaline phosphatase	58 U/L	55 U/L	40-115 U/L
B-natriuretic peptide	68 pg/mL	421.38	0-100 pg/mL
High sensitivity troponin	113 ng/L	756 ng/L	<78 ng/L
Total CK	85 U/L	289	38-176 U/L
Lactic acid	1.0 mmol/L	2.7 mmol/L	0–2.0 mmol/L
C-reactive protein	12.3 mg/dL	30.4 mg/dL	0.0-0.8 mg/dL
Procalcitonin	0.06 ng/mL	39.56 ng/mL	<0.5 ng/mL
Erythrocyte sedimentation rate	142 mm/hr	>150 mm/hr	0-20 mm/hr
Lactate dehydrogenase	432 U/L	794 U/L	122-222 U/L
Thyroid-stimulating hormone	3.25 uIU/mL		0.4-4.5 uIU/mL
Fibrinogen	575 mg/dL	775 mg/dL	200-393 mg/dL
Complete blood count			
WBC count	5.0 cells/µL	11.60 cells/µL	4,400-11,000 cells/µL
Hemoglobin	7.5 g/dL	8.4 g/dL	13.5-17.5 g/dL
Platelet count	45 cells/µL	44 cells/µL	150,000-450,000 cells/µL
Microbiology			
SARS Ag	Negative		Negative
SARS-CoV Naa	Positive	Positive	Negative
HIV		Negative	Negative
Rapid Influenza A/B Ag	Negative		Negative
AFB smear and culture		Negative	Negative
Blood culture	Negative	Negative	Negative
Urine Culture	Negative	Negative	Negative
Sputum culture	Negative	Negative	Negative

**Figure 1:**
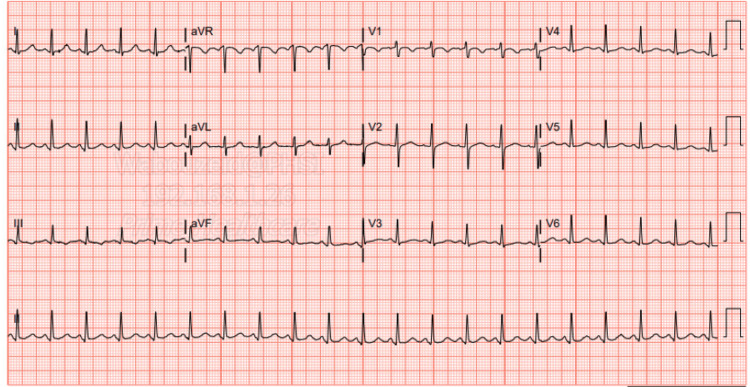
Admission EKG EKG on admission shows sinus tachycardia, with a heart rate of 130 bpm, and non-specific T wave inversion in leads III and V1 EKG: electrocardiogram

**Figure 2:**
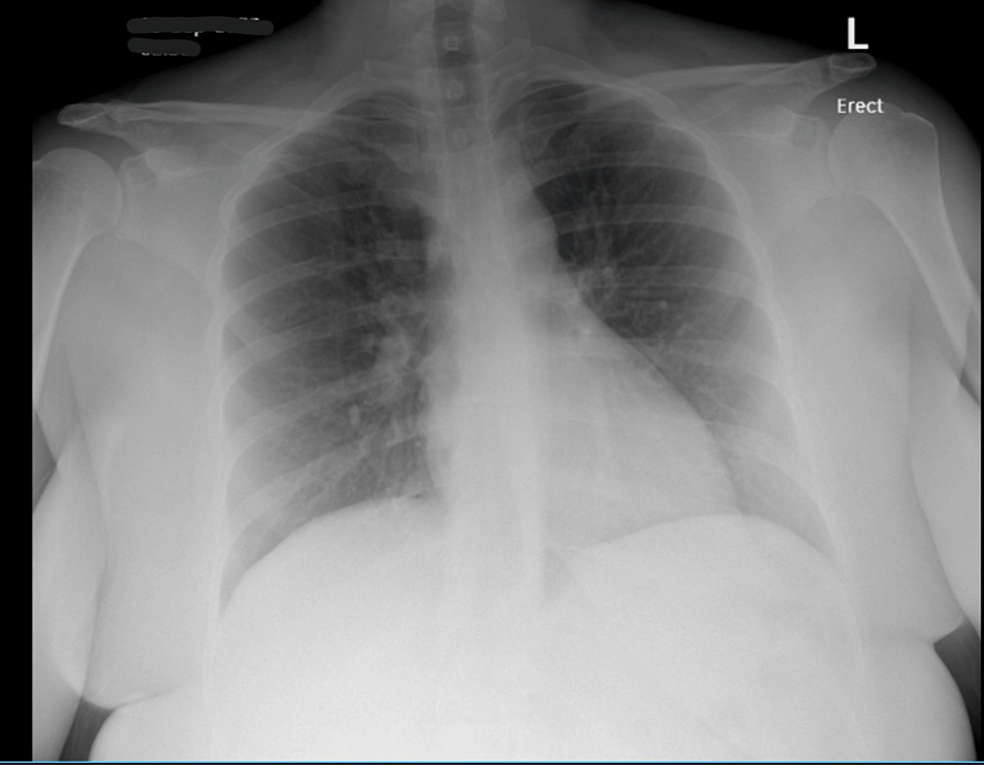
Admission chest X-ray anteroposterior view The chest radiograph on admission shows a normal cardiac silhouette

**Figure 3:**
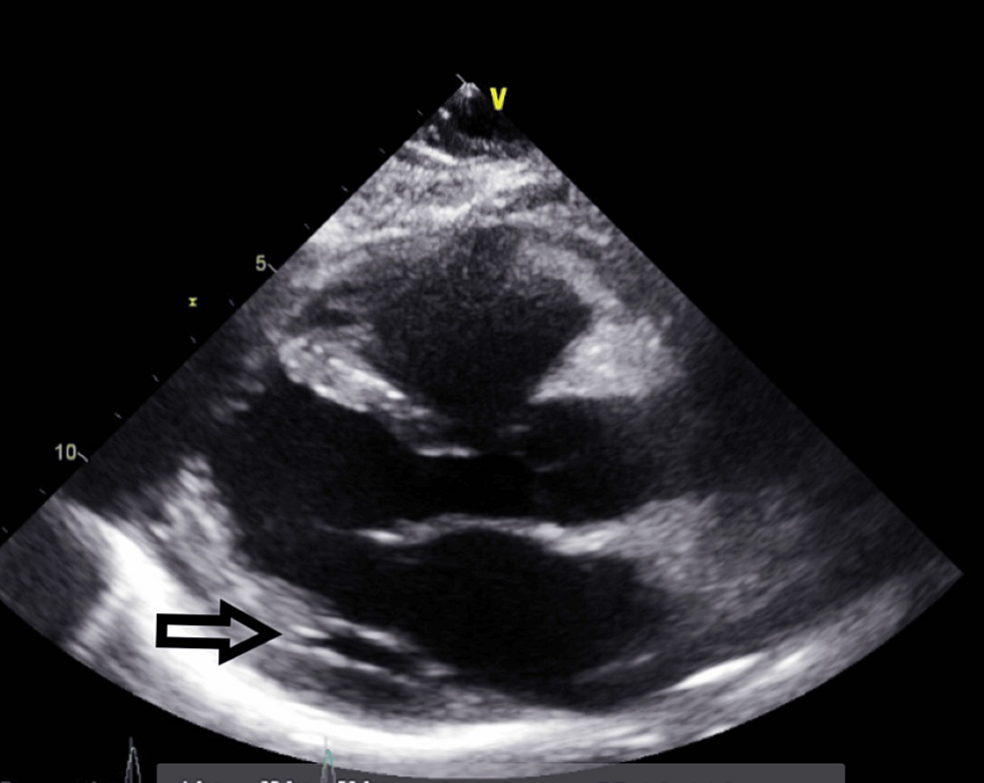
Transthoracic echocardiography - image 1 Transthoracic echocardiography on admission; parasternal long axis view shows minimal pericardial effusion

**Figure 4:**
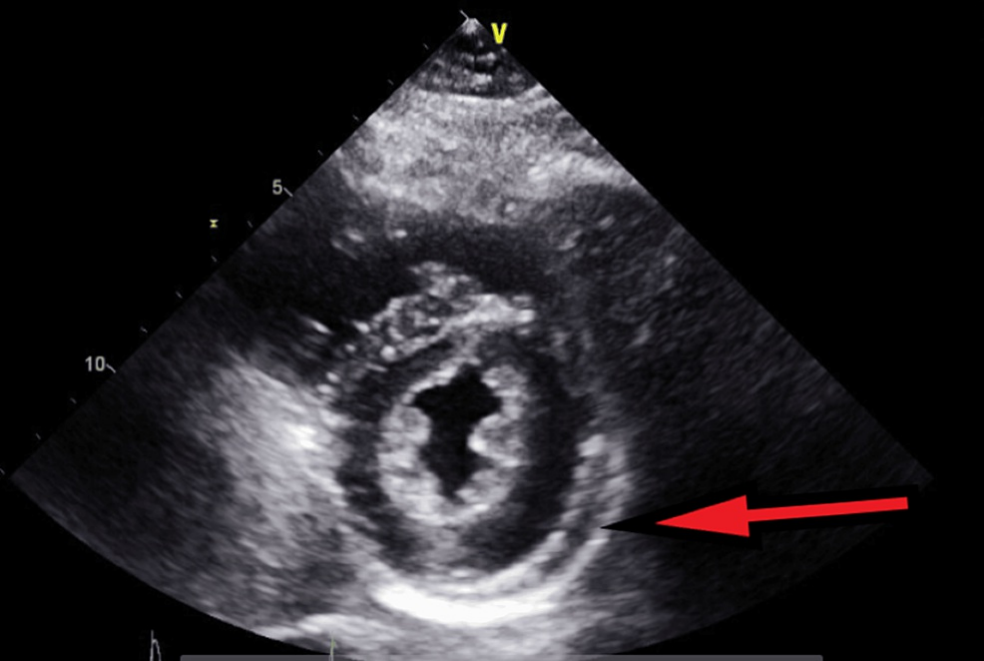
Transthoracic echocardiography - image 2 Transthoracic echocardiography on admission; parasternal short axis view shows a small rim of pericardial fluid collection indicative of the presence of minimal pericardial effusion

**Figure 5:**
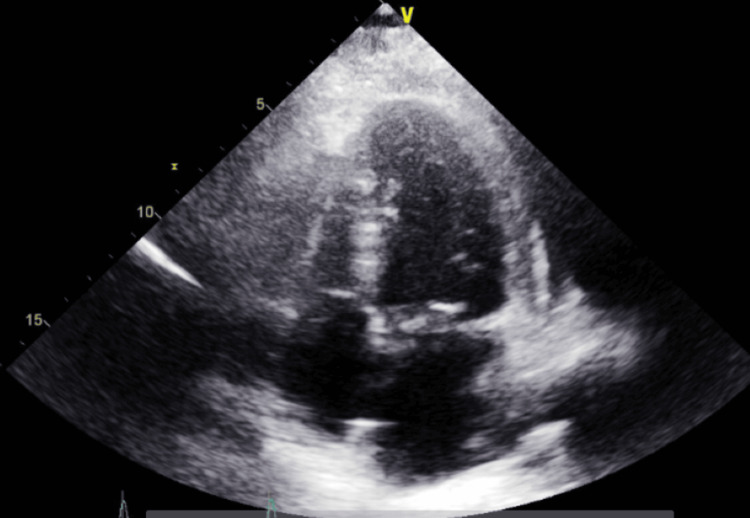
Transthoracic echocardiography - image 3 Transthoracic echocardiography on admission; apical 4-chamber view

**Figure 6:**
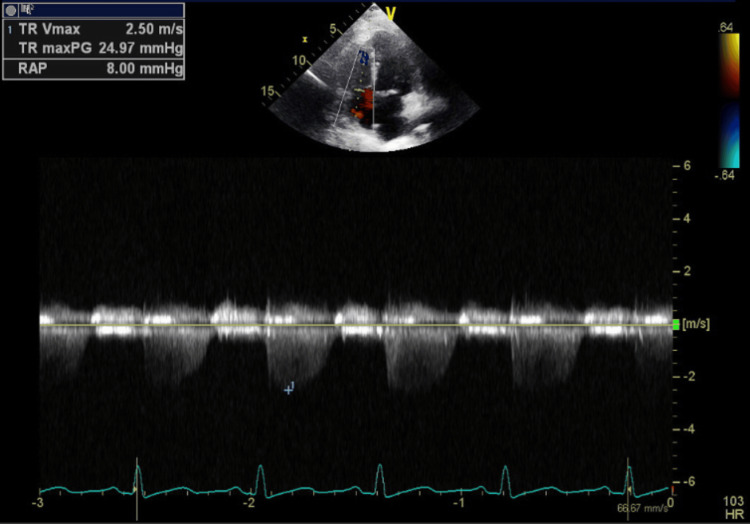
Transthoracic echocardiography - image 4 Transthoracic echocardiography on admission; apical 4-chamber view measuring TR Vmax at 2.50 m/s and RAP at 8 mmHg

The electrocardiogram (EKG) on admission showed sinus tachycardia with slight PR segment depression in lead II, but no ST elevations were seen otherwise (Figure 1). Transthoracic echocardiography (TTE) on admission showed a hyperdynamic ejection fraction of 70% with a trace pericardial effusion and no valvular abnormalities (Figures 3-6). Chest X-ray (CXR) did not show any significant abnormalities (Figure 2). The patient was started on colchicine; NSAIDs were avoided given anemia on presentation of unknown cause (hemoglobin of 7.5 g/dL).

**Figure 7:**
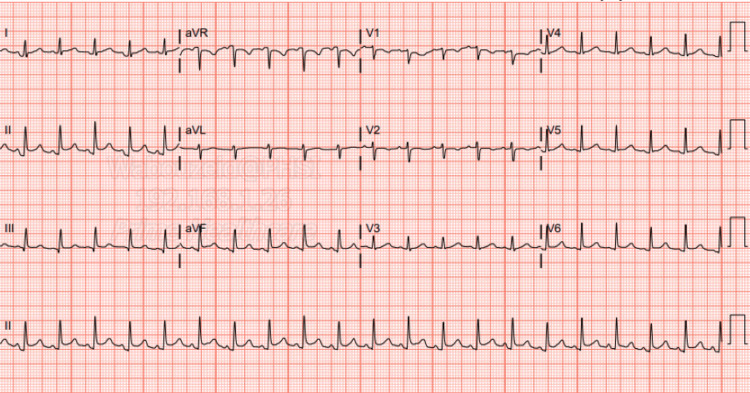
Follow-up EKG on the next day after admission Follow-up EKG on the next day shows sinus tachycardia, with PR interval depression noticed in leads I, II, aVF, V5, and V6 with electrical alternans sign noticed in lead II EKG: electrocardiogram

**Figure 8:**
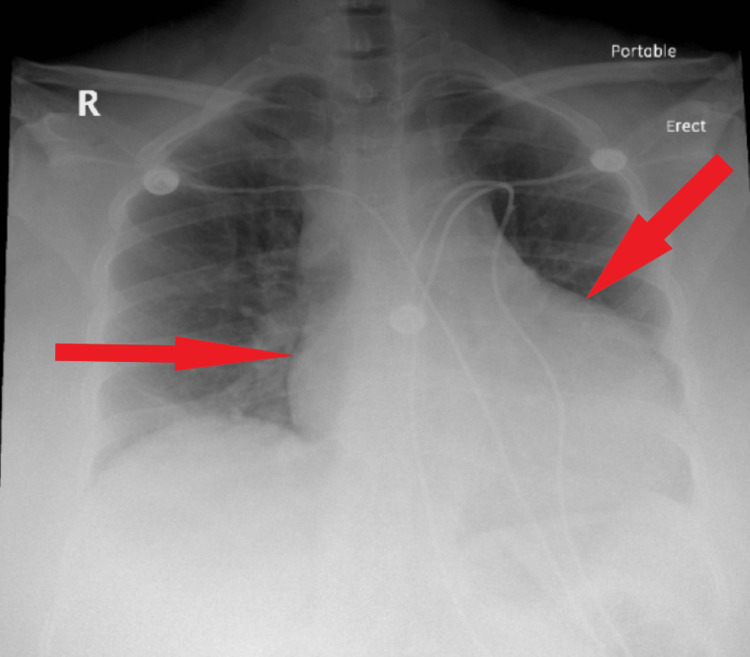
Follow-up chest X-ray posteroanterior view on the next day after admission The chest radiograph shows an enlarged cardiac silhouette, obscuring the lung hilum, indicative of pericardial effusion

**Figure 9:**
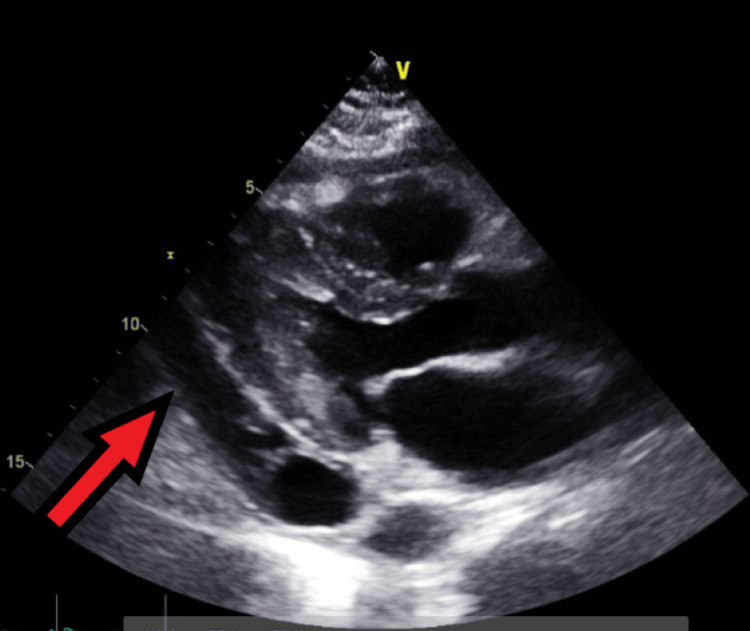
Follow-up transthoracic echocardiography on the next day after admission - image 1 Transthoracic echocardiography on the next day; parasternal long-axis view shows increased pericardial fluid collection (red arrow), indicative of large pericardial effusion

**Figure 10:**
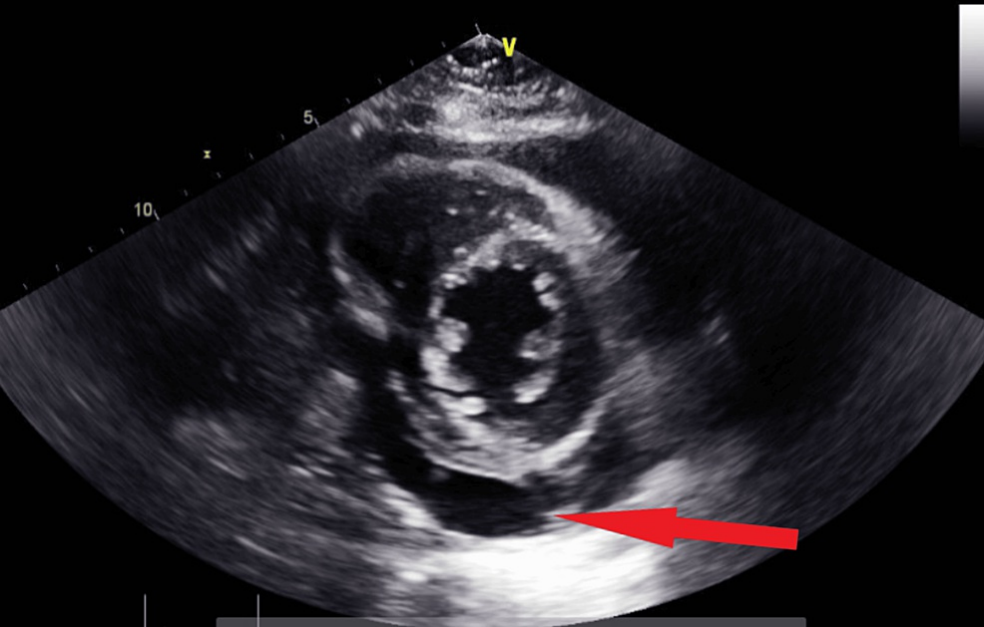
Transthoracic echocardiography on the next day after admission - image 2 Follow-up echocardiography on the next day; parasternal short axis view shows increased pericardial fluid collection (red arrow), indicative of large pericardial effusion

**Figure 11:**
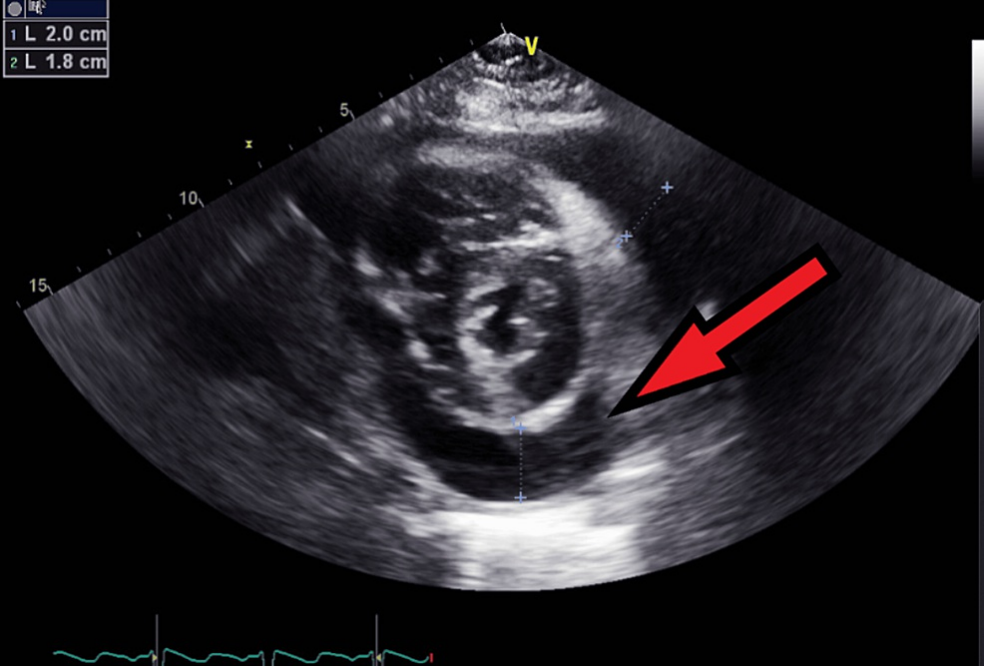
Transthoracic echocardiography on the next day after admission - image 3 Echocardiography on the next day; parasternal short axis view shows increased pericardial fluid collection (red arrow), measuring about 2 cm, indicative of large pericardial effusion

The next day, the patient started spiking fevers with a maximum temperature reaching 103 °F. She became tachypneic and tachycardic. A follow-up EKG was significant for sinus tachycardia with a heart rate of 120 bpm, with PR interval depression (Figure 7). A CXR was done and showed an enlarged cardiac silhouette compared to the previous CXR (Figure 8). An emergent bedside echocardiogram revealed a large pericardial effusion (Figures 9-11).

The patient developed Beck’s triad - hypotension, elevated jugular venous pressure, and muffled heart sounds. She therefore underwent an ultrasound-guided pericardiocentesis, which was performed successfully with the removal of 430 cc of serous fluid immediately, and the drain was kept in. The patient was evaluated clinically afterward and she reported feeling much better; there was improvement in her breathing and chest pain, and she could lay flat at this point. Her drain in place showed an additional 300 cc in the drainage bag with some fibrous components. The pericardial fluid component was found to be neutrophilic, with normal adenosine deaminase levels, and fluid cultures that were negative for bacterial growth.

A few hours later, the patient became more tachypneic and tachycardic again. Repeat bedside echocardiogram revealed reaccumulation of pericardial effusion with multiple septations compared to post-pericardiocentesis echocardiography, but there were no signs of tamponade. The pericardial drainage pigtail had stopped draining and there was concern that it might have been clogged, and hence it was manipulated. An attempt to place a larger catheter was made. Still, unfortunately, there was no more draining of pericardial fluid, which was most likely secondary to an abundance of fibrinous and coagulant material. The patient was intubated due to increased work of breathing with accessory muscle use and she was also started on treatment with IV glucocorticoids for the effusion, in addition to broad-spectrum antibiotics.

**Figure 12:**
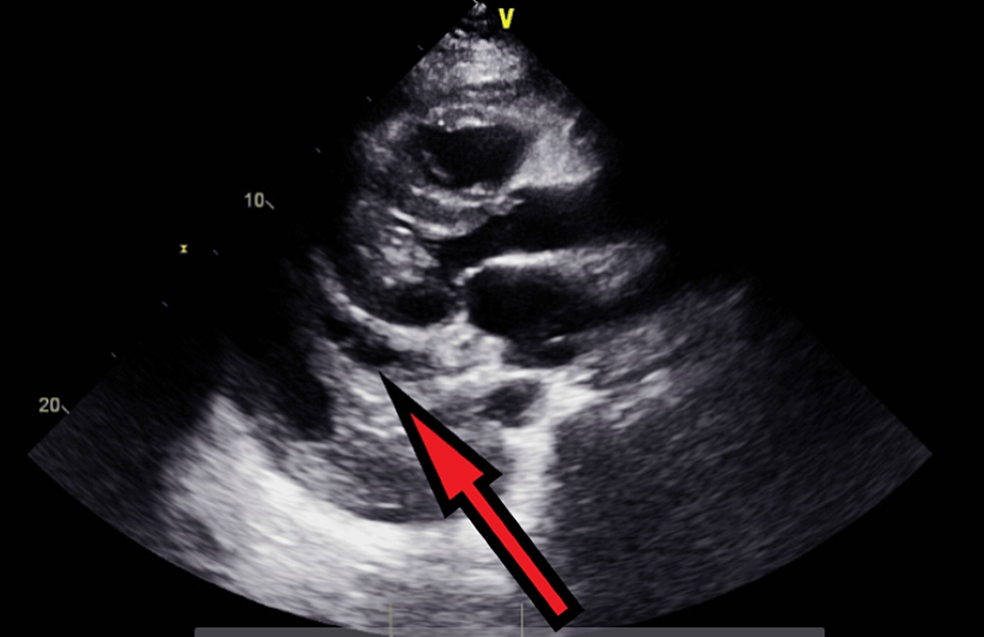
Transthoracic echocardiography post-pericardiocentesis - image 1 Transthoracic echocardiography post-pericardiocentesis; parasternal long axis view shows a decreased amount of pericardial fluid collection (red arrow)

**Figure 13:**
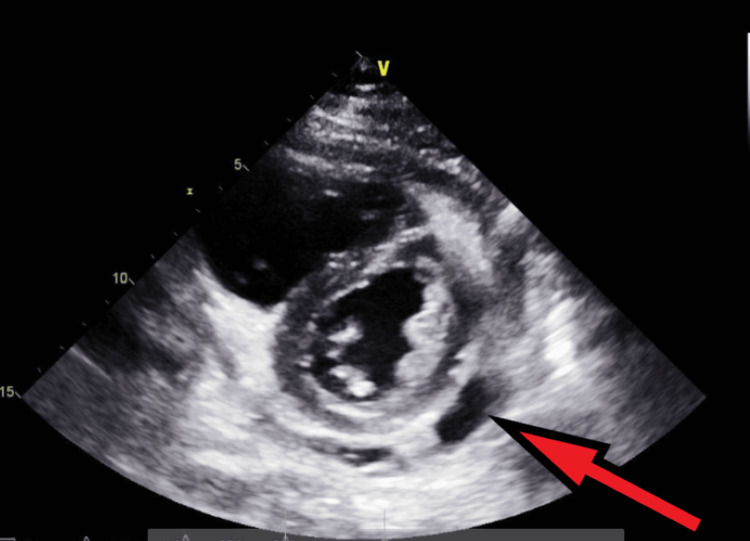
Transthoracic echocardiography post-pericardiocentesis - image 2 Transthoracic echocardiography post-pericardiocentesis; parasternal short axis view shows a decreased amount of pericardial fluid collection (red arrow) with the presence of some loculations

**Figure 14:**
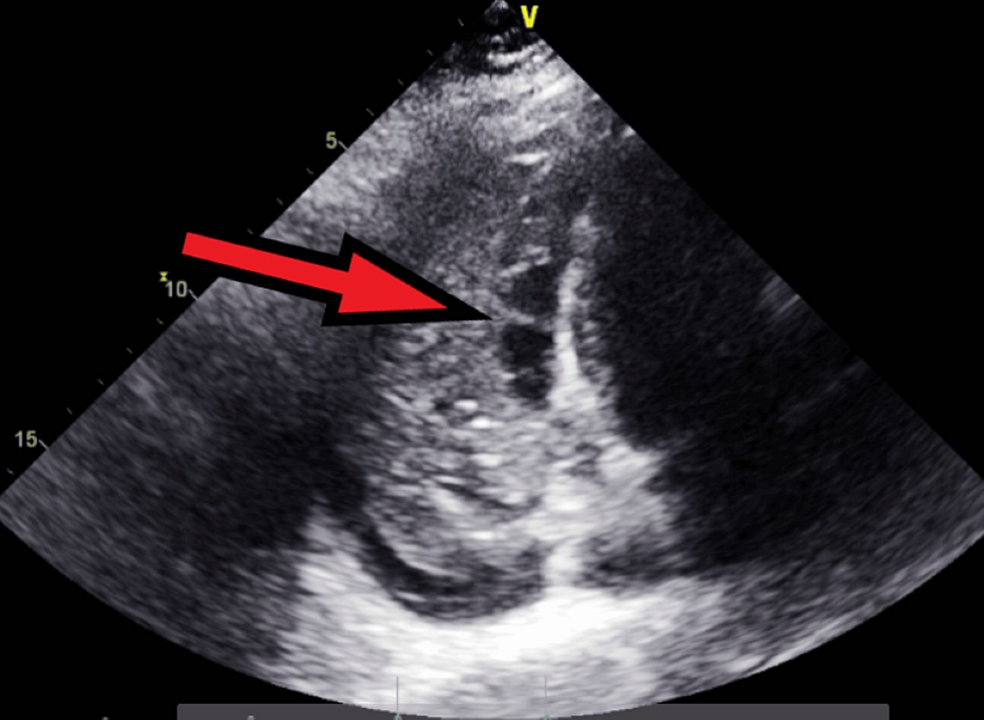
Transthoracic echocardiography post-pericardiocentesis - image 3 Transthoracic echocardiography post-pericardiocentesis; apical 4-chamber view showing multiple loculations (red arrow) with some remnant pericardial fluid collections

**Figure 15:**
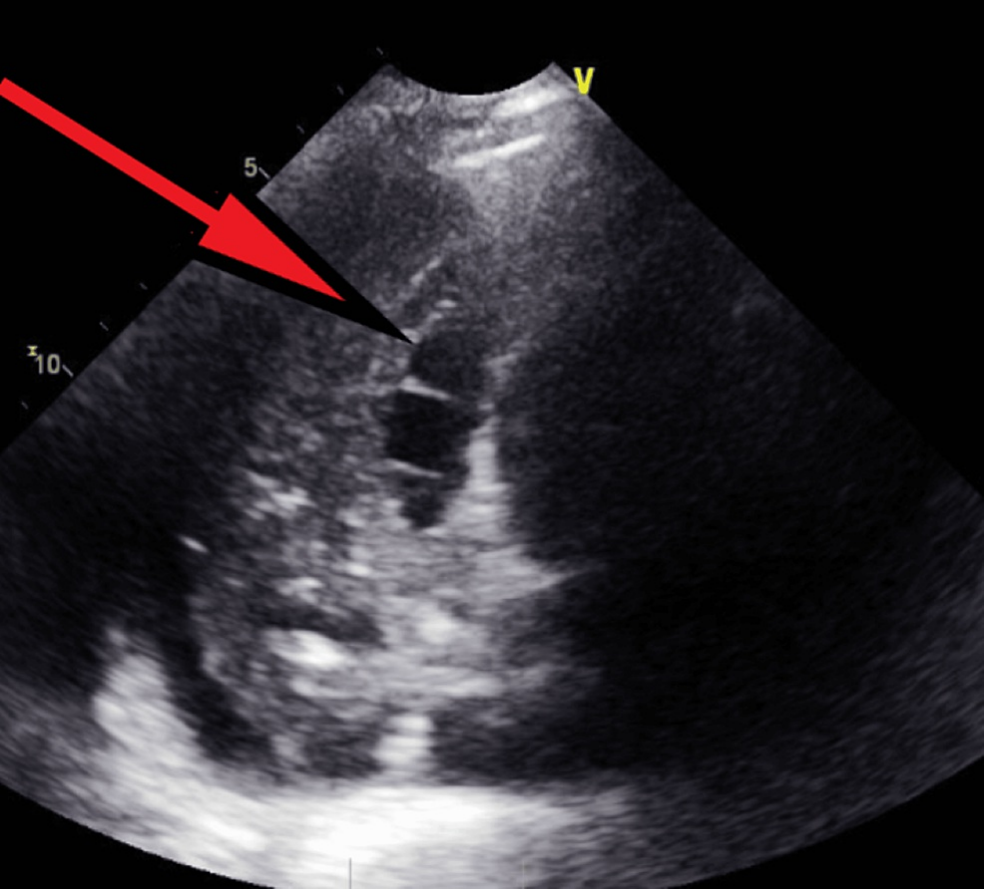
Transthoracic echocardiography post-pericardiocentesis - image 4 Transthoracic echocardiography post-pericardiocentesis; apical 4-chamber view showing another image of multiple loculations (red arrow) with some remnant pericardial fluid collections and the presence of septal bounce

**Figure 16:**
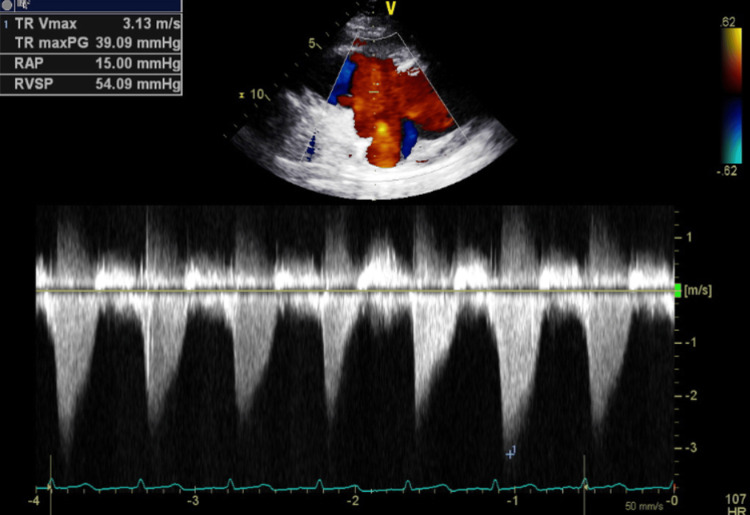
Transthoracic echocardiography post-pericardiocentesis - image 5 Transthoracic echocardiography post-pericardiocentesis; apical 4-chamber view measuring TR Vmax at 3.13 m/s, RAP at 15 mmHg, and RVSP at 54.09 mmHg, indicative of elevated right side pressure

**Figure 17:**
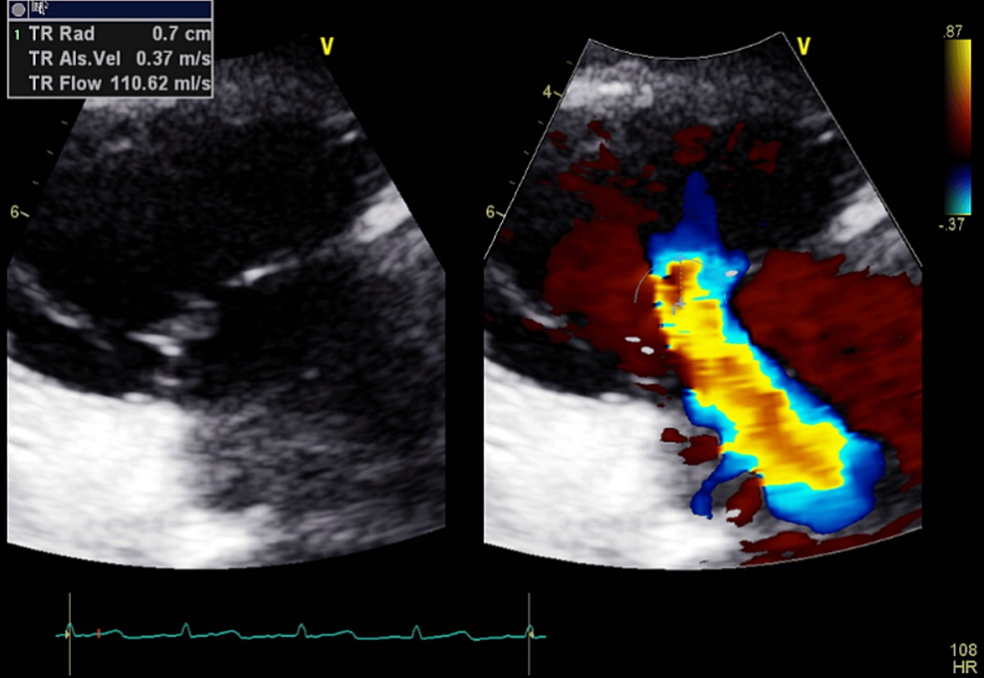
Transthoracic echocardiography post-pericardiocentesis - image 6 Transthoracic echocardiography post-pericardiocentesis; apical 4-chamber view measuring TR radius at 0.7 cm and TR flow at 119.62 ml/s, indicative of severe tricuspid regurgitation

An urgent TTE was done, which revealed severe right atrial and right ventricular enlargement with severe tricuspid regurgitation with a small to moderate septated pericardial effusion with multiple septations and septal bounce (Figures 12-17).

Based on the above-mentioned findings, any acute large pulmonary embolism leading to elevated right-sided pressures was ruled out, and the patient was therefore sent for a CT angiogram of the chest, which was negative. Elevated right heart pressures were confirmed with Swan-Ganz placement with RA pressure at 24/15 mmHg, RV pressure at 44/16 mmHg, PA pressure at 44/28 mmHg, and PCWP being normal at 12 mmHg.

**Figure 18:**
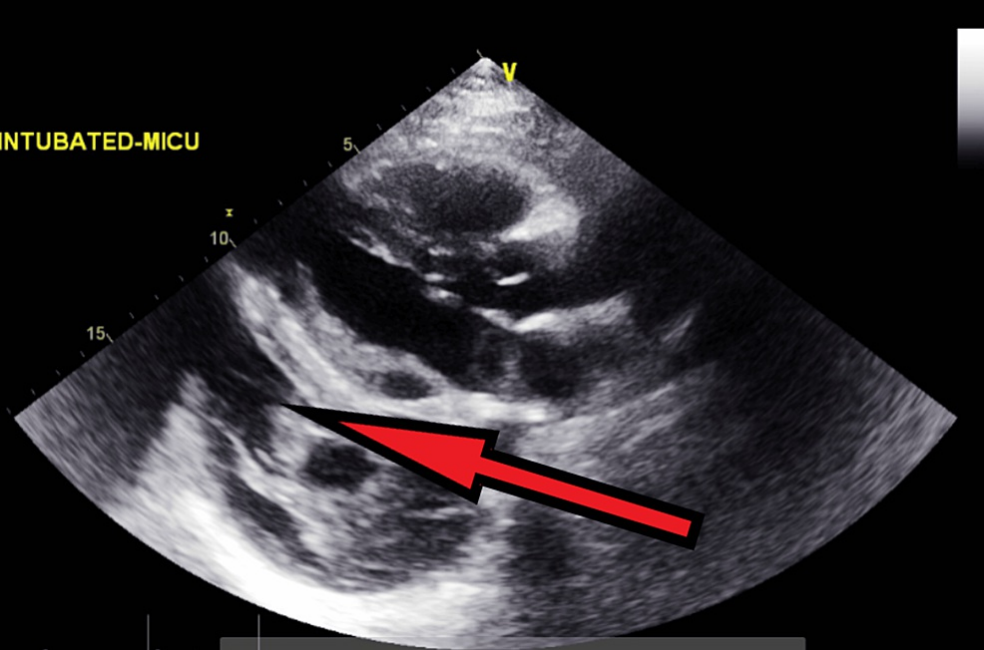
Follow-up transthoracic echocardiography after stabilization - image 1 Transthoracic echocardiography; long axis parasternal view shows improvement in the amount of pericardial fluid and hyperdynamic LV systolic function; LVEF: 65-70% LVEF: left ventricular ejection fraction

**Figure 19:**
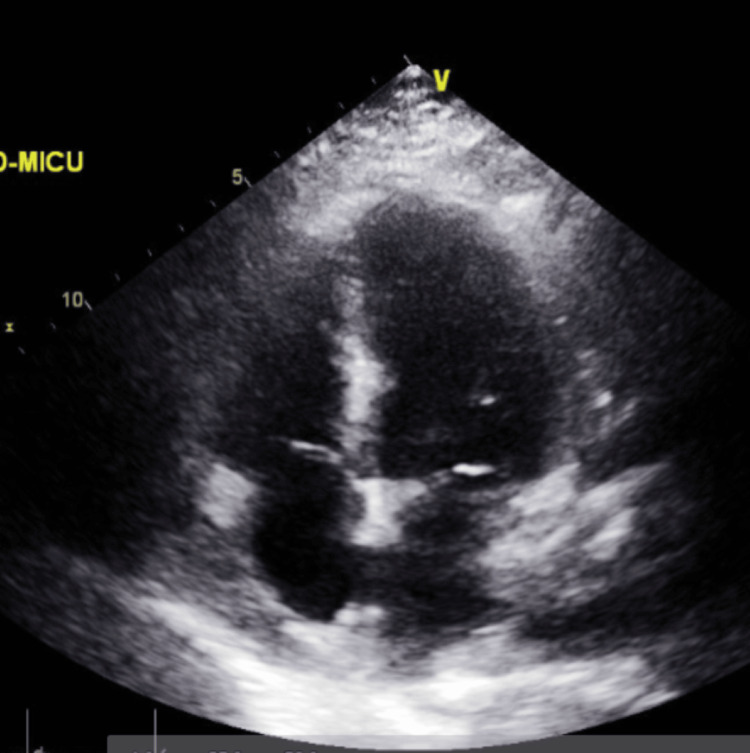
Follow-up transthoracic echocardiography after stabilization - image 2 Transthoracic echocardiography; apical 4-chamber view demonstrates the presence of minimal pericardial fluid collection

**Figure 20:**
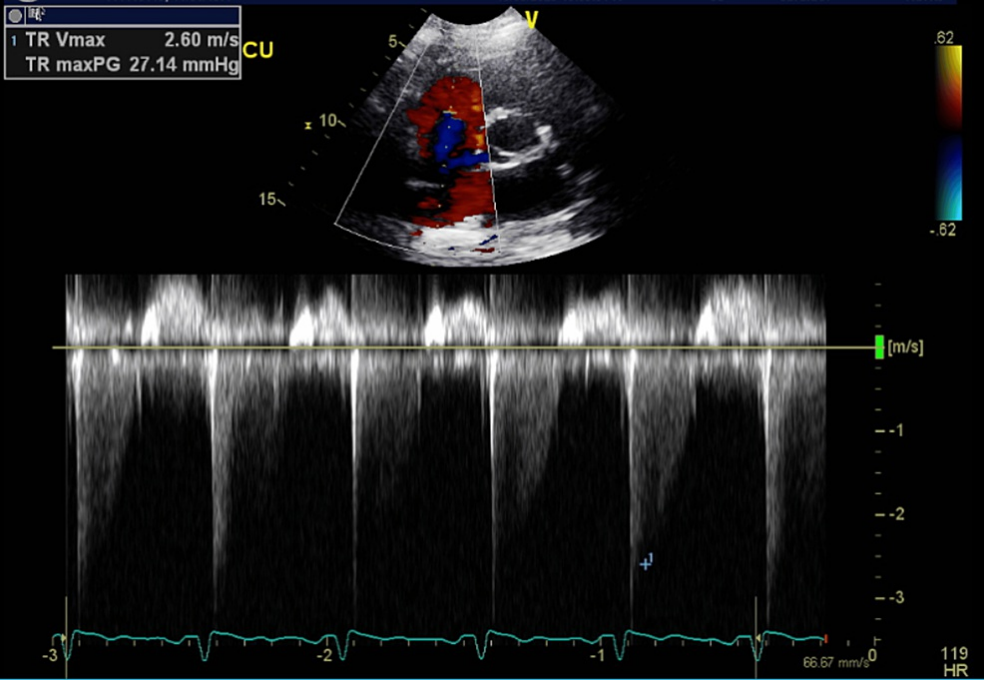
Follow-up transthoracic echocardiography after stabilization - image 3 Transthoracic echocardiography; apical 4-chamber view shows improvement in the degree of tricuspid regurge (TR Vmax: 2.60 m/s, TR maxPG: 27.14 mmHg)

**Figure 21:**
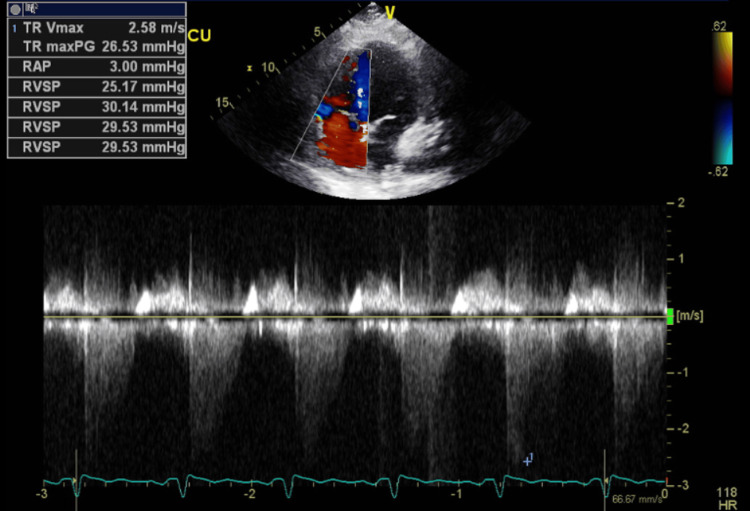
Follow-up transthoracic echocardiography after stabilization - image 4 Transthoracic echocardiography; apical 4-chamber view demonstrates improvement in right side pressures and the degree of tricuspid regurgitation (TR Vmax: 2.58 m/sec, TR maxPG: 26.53 mmHg, RAP: 3.00 mmHg, RVSP: ranging between 25.17 and 30.14 mmHg)

**Figure 22:**
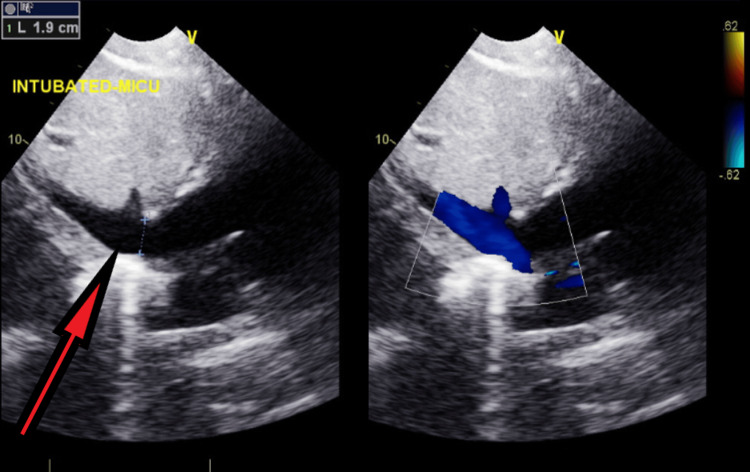
Follow-up transthoracic echocardiography after stabilization - image 5 Transthoracic echocardiography: sub-xiphoid view of IVC measuring 1.9 cm

After the patient went into right heart failure that progressed to cardiogenic shock, she was started on milrinone, norepinephrine, and vasopressin along with the continuation of steroids and broad-spectrum antibiotics, She remained intubated and sedated and hemodynamically supported by norepinephrine, milrinone, and vasopressin, with pulmonary artery pressure of 35 mmHg, PCWP of 15 mmHg, CVP of 20 cmH_2_o and SVO_2_ of 48%. TEE was repeated and showed mild improvement in the degree of tricuspid regurgitation (TR), and it was recommended to remove the Swan-Ganz catheter since it could be aggravating the TR. The catheter was removed and PA pressure, CVP, and SVO_2_ slowly improved; hence, vasopressors were weaned off after three days, and the patient remained hemodynamically stable off pressors. A follow-up echo a few days later showed hyperdynamic LV systolic function, LVEF at 65-70%, normal LV diastolic function, mild septal bounce, normal LA and RA size, mild TR regurgitation (significantly improved compared to most recent TEE), Normal RVSP, and normal IVC ( Figures 18-22).

Due to persistent bicytopenia despite the administration of steroids, IVIG, and multiple platelet transfusions, a bone marrow biopsy was done, which showed hypercellular bone marrow with NRAS and Tp-53 mutation, and findings consistent with acute myeloid leukemia (AML); the patient was started on cytarabine and idarubicin regimen, and the initial CBC showed pancytopenia, which improved later on with subsequent cycles.

## Discussion

We presented a case of a 45-year-old female who was admitted to the medical floor due to post-COVID-19 pericarditis with mild pericardial effusion that progressed to massive pericardial effusion and pericardial tamponade over 24 hours. After undergoing pericardiocentesis and placement of a pigtail catheter, the case was complicated by what we believe could have been a combination of ECP and PDS. This case illustrates the complexity of ECP, a rare condition that combines features of pericardial effusion and constrictive physiology. It highlights the potential role of viral infections in triggering ECP and the challenges in its diagnosis and management, including the risk of PDS.

Acute pericarditis, pericardial effusion, cardiac injury leading to myocarditis, myocardial infarction, left or right ventricular dysfunction, arrhythmias, and congestive heart failure are commonly reported cardiovascular complications post-COVID-19 infection. Acute ECP is considered a rare manifestation of COVID-19 infection, especially in the absence of pulmonary or myocardial injury; hence, diagnosing ECP can be challenging due to its diverse clinical presentations and the need for multimodal imaging and hemodynamic assessments. In this case, the initial echocardiogram revealed a hyperdynamic ejection fraction, which can be misleading in the context of constrictive pericarditis. This highlights the importance of a comprehensive diagnostic workup, including serial imaging, and consideration of alternative diagnosis besides a high index of suspicion to ensure early diagnosis and treatment as diagnosing these conditions can be challenging. Furthermore, early appropriate treatment is essential for favorable outcomes. Pericardiocentesis is the treatment of choice for patients with symptomatic pericardial effusion. Although symptomatology and hemodynamic abnormalities typically improve dramatically after pericardiocentesis, a small subset of patients may fail to show resolution of symptoms or may even deteriorate after pericardiocentesis. Such a scenario is usually associated with the development of ECP [7-14].

ECP post-pericardiocentesis is rare and characterized by the coexistence of tense pericardial effusion and constriction of the heart by the visceral pericardium, which can be diagnosed based on the persistent elevation in right atrial pressure via invasive hemodynamic assessment after restoring intrapericardial pressure via pericardiocentesis. Invasive hemodynamic assessment by cardiac catheterization is the gold standard for diagnosing ECP but it can also be diagnosed after pericardiocentesis by visualizing abnormal ventricular septal motion or measuring the dissociation of intrathoracic and intracardiac pressures using echo-Doppler. It may also be diagnosed by utilizing T2-weighted cardiac MRI, which can delineate the pericardial thickening further and distinguish between edema, inflammation, and fibrosis. ECP may also help diagnose concomitant myocarditis by demonstrating typical features including myocardial injury (hyperemia, necrosis, scarring) in T1-weighted and myocardial edema in T2-weighted imaging [13-15]. ECP post-pericardiocentesis usually resolves either spontaneously or with medical management using NSAIDs, colchicine, or steroids for pain and inflammation. Pericardiectomy is reserved for patients refractory to adequate anti-inflammatory therapy. Unfortunately, it has been reported that up to 16% of patients may continue to have symptoms, eventually requiring extensive pericardiectomy [15,16].

On the other hand, rapid removal of pericardial fluid may result in PDS, which is also an uncommon complication of pericardial drainage that has high morbidity and mortality and usually results from rapid removal of the fluid in cases of cardiac tamponade compressing RV. This, in turn, leads to an increase in venous return and causes significant RV dilatation, which leads to TR and decreased left-sided volume, decreased cardiac output, and subsequent acute heart failure and pulmonary edema. This usually presents with a paradoxical worsening of the patient’s hemodynamics after initial improvement. The onset of this syndrome has been reported to range from immediate to 48 hours post-pericardiocentesis in the form of LV failure and cardiogenic shock in up to 40% of the cases, with pulmonary edema without shock in 29%, with shock associated with biventricular failure in 20%, and with shock associated with RV failure and non-cardiogenic pulmonary edema in 11%. PDS usually requires supportive management as the improvement of ventricular function is expected, in the form of inotropic support, aggressive heart failure treatment with pressors and diuretics, and hemodynamic support devices, such as an intra-aortic balloon pump [17-19].

Although there are no clear guidelines or recommendations to prevent PDS, a staged or gradual drainage of pericardial fluid to minimize abrupt hemodynamic shifts should be encouraged. This can be achieved by the removal of the minimal amount that will result in the resolution of the cardiac tamponade physiology with the placement of prolonged pericardial drainage to achieve a slow and gradual removal of additional pericardial fluid, which can be removed when there is a daily fluid return below 30-50 mL. The use of vasopressors and inotropes may also be necessary to support circulation during pericardiocentesis, as demonstrated in this case [20].

## Conclusions

Acute pericarditis and pericardial effusion are well-known cardiac manifestations of COVID-19 infection. ECP is a rare but clinically significant condition that requires a high index of suspicion for prompt diagnosis and management. Close monitoring and serial echocardiography are required for the quantification of the pericardial fluid and early detection of signs of cardiac tamponade, which necessitates pericardiocentesis, and it is advised to drain the amount just enough to reverse the tamponade physiology, followed by the placement of a drain for slow drainage of the rest of pericardial fluid to avoid PDS. This report not only highlights the complex nature of ECP but also offers insights into the interplay between recent COVID-19 infection and the emergence of PDS.
